# Enhanced biological fixation of methane for microbial lipid production by recombinant *Methylomicrobium buryatense*

**DOI:** 10.1186/s13068-018-1128-6

**Published:** 2018-05-04

**Authors:** Qiang Fei, Aaron W. Puri, Holly Smith, Nancy Dowe, Philip. T. Pienkos

**Affiliations:** 1 School of Chemical Engineering and Technology, Xi’an Jiaotong University, Xi’an, China; 2 National Bioenergy Center, National Renewable Energy Laboratory, Golden, CO USA; 3 Department of Chemical Engineering, University of Washington, Seattle, WA USA

**Keywords:** Methane biofixation, Methanotrophic bacteria, Membrane lipids, High cell density culture, Bioconversion process, Metabolic engineering

## Abstract

**Background:**

Due to the success of shale gas development in the US, the production cost of natural gas has been reduced significantly, which in turn has made methane (CH_4_), the major component of natural gas, a potential alternative substrate for bioconversion processes compared with other high-price raw material sources or edible feedstocks. Therefore, exploring effective ways to use CH_4_ for the production of biofuels is attractive. Biological fixation of CH_4_ by methanotrophic bacteria capable of using CH_4_ as their sole carbon and energy source has obtained great attention for biofuel production from this resource.

**Results:**

In this study, a fast-growing and lipid-rich methanotroph*, Methylomicrobium buryatense* 5GB1 and its glycogen-knock-out mutant (AP18) were investigated for the production of lipids derived from intracellular membranes, which are key precursors for the production of green diesel. The effects of culture conditions on cell growth and lipid production were investigated in high cell density cultivation with continuous feeding of CH_4_ and O_2_. The highest dry cell weight observed was 21.4 g/L and the maximum lipid productivity observed was 45.4 mg/L/h obtained in batch cultures, which corresponds to a 2-fold enhancement in cell density and 3-fold improvement in lipid production, compared with previous reported data from cultures of 5GB1. A 90% enhancement of lipid content was achieved by limiting the biosynthesis of glycogen in strain AP18. Increased CH_4_/O_2_ uptake and CO_2_ evaluation rates were observed in AP18 cultures suggesting that more carbon substrate and energy are needed for AP18 growth while producing lipids. The lipid produced by *M. buryatense* was estimated to have a cetane number of 75, which is 50% higher than biofuel standards requested by US and EU.

**Conclusions:**

Cell growth and lipid production were significantly influenced by culture conditions for both 5GB1 and AP18. Enhanced lipid production in terms of titer, productivity, and content was achieved under high cell density culture conditions by blocking glycogen accumulation as a carbon sink in the strain AP18. Differences observed in CH_4_/O_2_ gas uptake and CO_2_ evolution rates as well as cell growth and glycogen accumulation between 5GB1 and AP18 suggest changes in the metabolic network between these strains. This bioconversion process provides a promising opportunity to transform CH_4_ into biofuel molecules and encourages further investigation to elucidate the remarkable CH_4_ biofixation mechanism used by these bacteria.

## Background

**Fig. 1:**
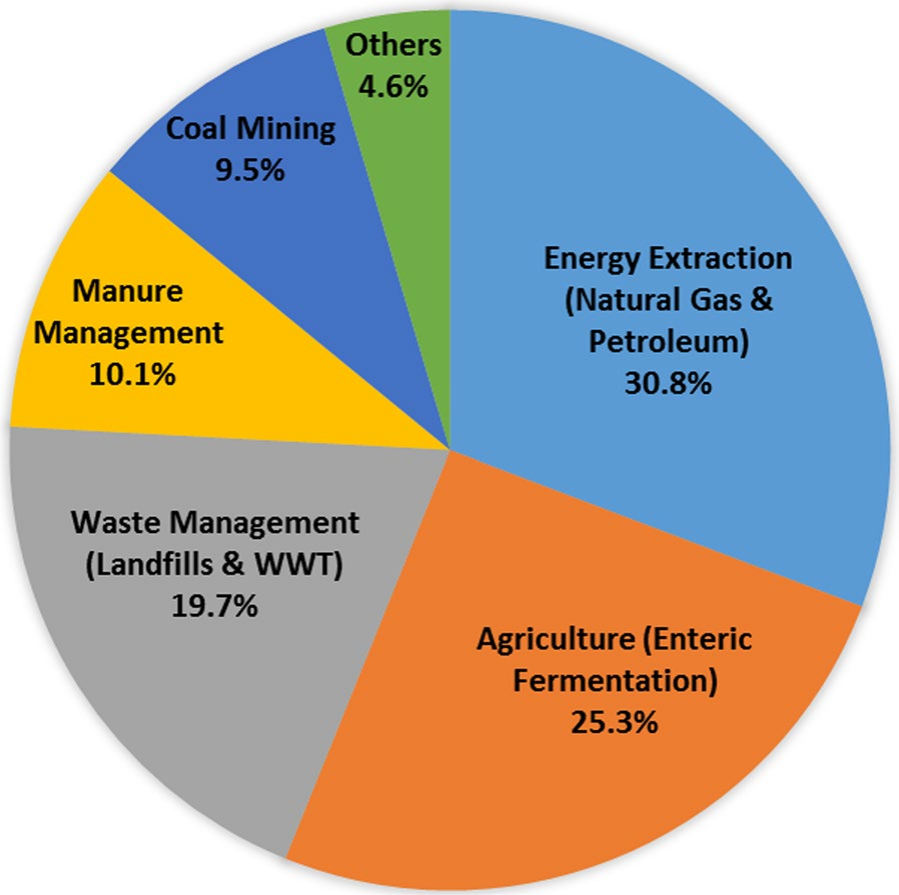
Methane emission by sources from 1990 to 2015 in the US (data taken from the report of US Environmental Protection Agency: Inventory of US Greenhouse Gas Emissions and Sinks: 1990–2015). *WWT* wastewater treatment

Methane (CH_4_) is the simplest and most energy dense alkane with a specific energy of 55 MJ/kg. Only 36% of CH_4_ released into the atmosphere is contributed by natural geological activities [1], while most of the rest is due to human activities (Fig. 1), such as energy extraction, agriculture, and waste management [2]. Since 2008, the development of shale gas technologies in the US has reduced the cost of natural gas significantly, making CH_4_ (the major component of natural gas) a potential alternative carbon source for bioconversion processes compared with other high-price raw material sources or edible feedstocks [3]. However, more than 5.4 quads (1.47 × 105 million cubic meters) of natural gas have been flared annually as a wasted resource at oil production sites around the globe [4, 5]. Therefore, it is urgent to explore alternative approaches to use CH_4_ more efficiently and economically. Biological fixation of CH_4_ by microorganisms has been attracting great attention and is a promising strategy to increase the value of natural gas [6] as well as another major source of CH_4_, biogas [7].

Methanotrophic bacteria, which were discovered in 1906 [8] and then characterized beginning in the 1970s [9], are a group of bacteria capable of utilizing CH_4_ as their sole carbon and energy source. Although some methanotrophs are able to grow in anaerobic environments, only aerobic methanotrophs have been isolated in pure culture and are genetically tractable. These organisms can oxidize CH_4_ to methanol and beyond for both catabolism and anabolism and therefore have the ability to serve as a production platform for the conversion of CH_4_ into bio-based products [10–12].

**Fig. 2:**
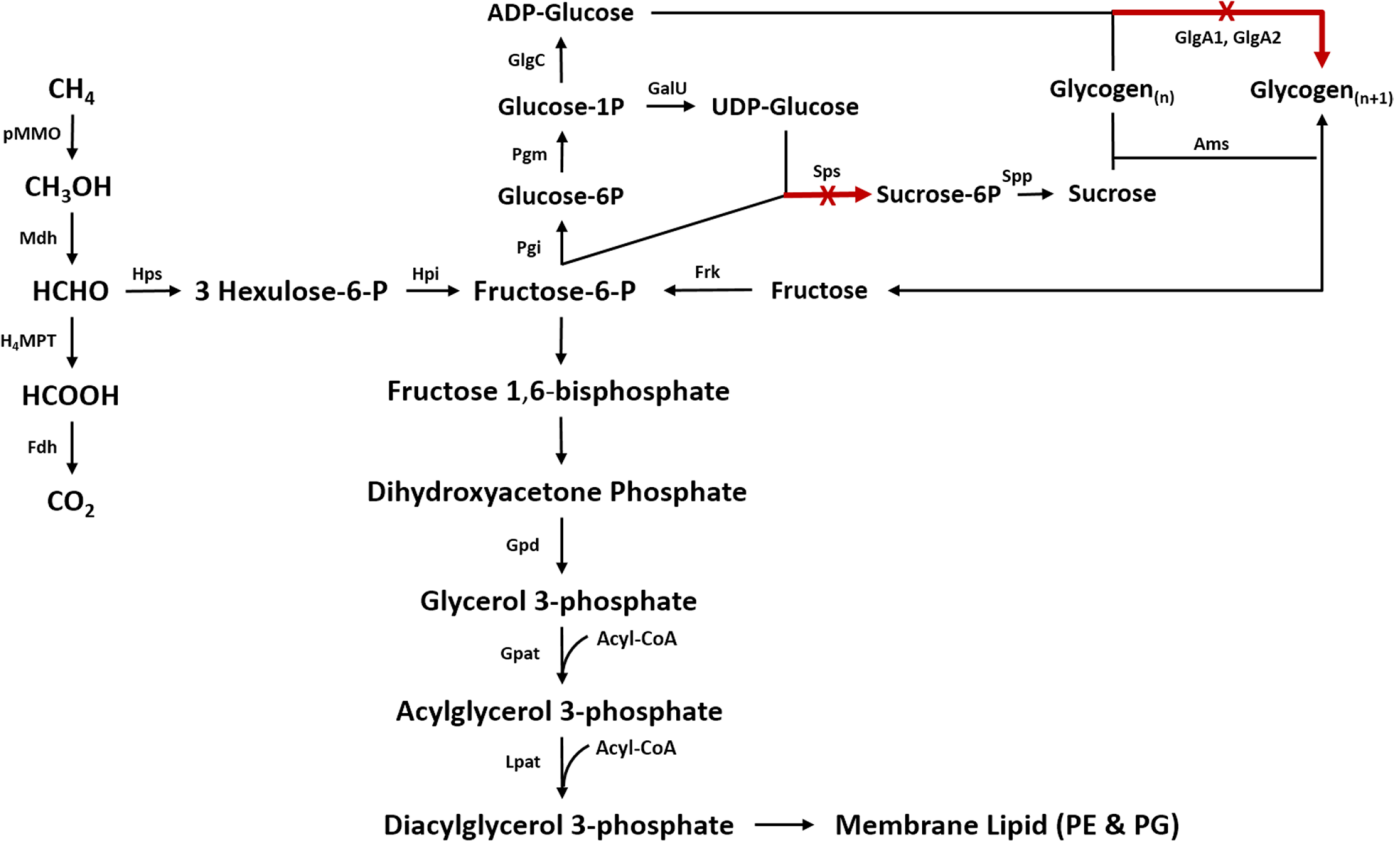
Pathways for the biosynthesis of membrane lipids and glycogen in *M. buryatense* based on ribulose monophosphate (RuMP) cycle. *pMMO* particular methane monooxygenase, *Mdh* methanol dehydrogenase, *H4MPT* methylene tetrahydromethanopterin, *Fdh* formate dehydrogenase, *Hps* hexulose-phosphate synthase, *Hpi* hexulose-phosphate isomerase, *Gpat* glycerol-3-phosphate acyltransferase, *Lpat* acylglycerol-3-phosphate acyltransferase, *Pgi* glucosephosphate isomerase, *Frk* fructokinase, *Sps* sucrose-6-phosphate synthase, *Pgm* phosphoglucomutase, *Spp* sucrose-6-phosphate phosphatase, *GalU* glucose-1-phosphate adenylyltransferase, *Ams* amylosucrase, *GlgA* glycogen synthase

Biofuels are an important component of a global effort to replace fossil fuels with renewable sources of energy due to enormous demand in terms of environmental issues, sustainable considerations, and greenhouse gas emissions, and methane-based biological gas to liquids (Bio-GTL) technologies could contribute to the various biofuel strategies under development [13, 14]. In 2013, a program announced by the Advanced Research Projects Agency-Energy (ARPA-E) of the US Department of Energy, named Reducing Emissions using Methanotrophic Organisms for Transportation Energy (REMOTE), was initiated to accelerate the development of economic processes for bioconversion of CH_4_ into liquid fuels [6]. One biocatalyst selected for this work was the methanotroph *Methylomicrobium buryatense,* which grows robustly at a range of pH and salinity levels [15]. *M. buryatense* is an aerobic gammaproteobacterium that oxidizes methane to methanol and then formaldehyde, which is subsequently incorporated into cellular biomass via the ribulose monophosphate cycle (Fig. 2). A set of genetic tools have been developed for *M. buryatense* [16, 17], and have been used to engineer this microorganism to produce lactic acid [18] as well as increase fatty acid levels during growth on CH_4_ [19, 20].

Fatty acids and glycerolipids such as phosphatidyl ethanolamine (PE) and phosphatidyl glycerol (PG) are included among the list of cellular components produced during growth on CH_4_. These are incorporated into membranes, which can be upgraded to diesel blend stocks for biofuel through a catalytic process known as hydrodeoxygenation [6]. A lab-scale demonstration of an integrated biorefinery process was successfully developed recently, in which process membrane lipids produced by *M. buryatense* were converted into green diesel [21]. Gilman et al. investigated effects of CH_4_ and O_2_ limitation on *M. buryatense* growth and lipid production in continuous cultures, and discovered that oxygen-limited condition provided the highest dry cell weight (DCW) of 0.79 g/L and lipid content of 10.7% (w/w) [22]. Although the production performance of that research in terms of cell density and lipid productivity was low compared with oleaginous yeasts growing on sugars or microalgae growing photosynthetically with CO_2_, the results provided the basis for improvement in the work described here.

In order to provide more insights into using *M. buryatense* in an industrial setting, this study summarizes our efforts to achieve high lipid titer and productivity in high cell density cultures. Additionally, a mutant strain (*M. buryatense* AP18) in which the carbon sink of glycogen was eliminated was constructed and tested under the same conditions. The cell growth and lipid production from both strains were studied and compared in order to elucidate the influence of culture conditions on lipid production, gas uptake/evolution rates, and glycogen accumulation in batch cultures. Finally, the fatty acid compositions of lipids produced by 5GB1 and AP18 were analyzed and characterized for diesel fuel production.

## Methods

### Strain, media, and chemicals

The frozen stocks of *M. buryatense* 5GB1 and its mutants were preserved under − 80 °C with DMSO as described previously [15]. For seed cultures, *M. buryatense* 5GB1 and AP18 were grown at pH 9.0 ± 0.2 using a defined NMS2 medium [17] containing the following, per liter of distilled water: KNO_3_ 2 g, MgSO_4_·7H_2_O 1 g, CaCl_2_·6H_2_O 0.02 g, NaCl 7.5 g, phosphate solution (KH_2_PO_4_ 5.44 g/L and Na_2_HPO_4_ 10.73 g/L) 20 mL, carbonate solution (1 M NaHCO_3_ 700 mL and 1 M Na_2_CO_3_ 300 mL) 50 mL, and trace element solution 1 mL [23]. CH_4_ of 99.97% purity (United States Welding Inc, Denver, USA) was used as the sole carbon source for all cultures. Due to safety considerations, a gas mixture with 20% CH_4_ and 80% air (v/v) was used for this study to avoid the flammable zone [24]. A continuous gas delivery system was used to supply the gas mixture for all CH_4_ cultures. Unless otherwise stated, all chemicals were purchased from Sigma-Aldrich Co. LLC (St. Louis, MO, US).

### Plasmid and strain construction

Strains and plasmids used in this study are listed in Table 1. The plasmid pAB2 for creating an unmarked deletion of the sucrose-phosphate synthase gene in *M. buryatense* (*sps*, MaGE [25] locus tag MBURv2_130613) was constructed using Gibson Assembly [26]. The primers used are listed in Table 2, and the vector backbone of a kanamycin-resistant version of pCM433 was amplified from the plasmid pAWP45 [17]. The PCR template for amplification of the *sps* flanking regions was genomic DNA extracted from *M. buryatense* 5GB1 using the DNeasy blood and tissue kit number 69504 (Qiagen, Hilden, Germany). To construct the triple knock out strain AP18, pAB2 was introduced into the strain 5GB1 ∆*glgA1* ∆*glgA2* via conjugation and sucrose counterselection was performed as previously described [17]. In brief, *M. buryatense* 5GB1 ∆*glgA1* ∆*glgA2* was spread onto an NMS2 mating plate and grown overnight. An equal volume of *E. coli* S17-1 λpir donor cells containing pAB2 was then added to the plate for conjugation, and the resulting mixture was incubated at 30 °C for 2 days. The mixture of cells was then spread onto NMS2 plates containing kanamycin (50 μg/mL) to select for transconjugants, which were subsequently purified from the donor via passaging on NMS2 plates containing rifamycin (50 μg/mL). For counterselection, double crossovers were selected by plating kanamycin-resistant transconjugants (single crossovers) onto NMS2 containing 2.5% sucrose and rifamycin. Colonies were then checked for kanamycin sensitivity and genotyped via PCR, using either colonies as the PCR template or DNA extracted with DNeasy blood and tissue kit (#69504 Qiagen, Hilden, Germany). Subsequently, this strain was cured of the native 80-kb plasmid using vector pAWP93, in order to enable this strain to accept small plasmids for heterologous expression in future studies as previously described [17].

**Table 1:** Strains and plasmids used in this study

Strain/plasmid	Description	References
Strains		
*E. coli* TOP10	F–*mcrA* Δ (*mrr*-*hsdRMS*-*mcrBC*) Φ80*lacZ*ΔM15 Δ*lacX74 recA1 araD139* Δ (*ara leu*) *7697 galU galK rpsL* (Str^R^) *endA1 nupG*	Invitrogen
*E. coli* S17-1 λpir	Donor strain. Tp^R^ Sm^R^ *recA*, *thi, pro, hsd* (R^-^M^+^) RP4: 2-Tc:Mu: Km Tn7 λ*pir*	[27]
*M. buryatense* 5GB1	Moderately haloalkaliphilic methanotroph	[17]
*M. buryatense* 5GB1 ∆*glgA1* ∆*glgA2*	*M. buryatense* 5GB1 ∆*glgA1* ∆*glgA2*	[17]
AP18	*M. buryatense* 5GB1C [17] ∆*glgA1* ∆*glgA2* ∆*sps*	This study
Plasmids		
pAWP93	pCM433kanT containing flanks to knock out *repA*-*repB* locus of native plasmid.	[17]
pAB2	Kanamycin-resistant variant of pCM433 containing flanks to knock out *sps*	This study

**Table 2:** Primers used in this study

Primer name	Sequence (5′–3′)	Description
AP186_pCM433kanT_fwd1	ATGTGCAGGTTGTCGGTGTC	For amplifying the backbone of the KanR version of the sucrose counterselection plasmid pCM433 [17]
AP187_pCM433kanT_rev1	TGGTAACTGTCAGACCAAGTTTACTC	
AP110_spsKO_UP_Fwd	ATTGGTACCATGGATGCATATGCTGCAGCTACGCTGCTCTAAATACCTTG	For amplifying flanks to knock out sps (MaGE locus tag MBURv2_130613) using plasmid pAB2
AP111_spsKO_UP_Rev	TCTGCTCTCAGTCACGATCGTACTCACTTCAGCTG	
AP112_spsKO_DWN_Fwd	GAAGTGAGTACGATCGTGACTGAGAGCAGAATTATGACTG	
AP113_spsKO_DWN_Rev	AGTGAGCTCACCGGTTAACACGCGTACGTACCTTCGGCCAAATACAACGA	

Homology regions used for Gibson assembly are underlined

### Culture conditions

A loop of colonies taken from an agar plate was used to inoculate seed cultures in defined NMS2 medium in a 300-mL spinner bottle (Bellco Glass, Inc., Vineland, NJ) at 30 °C and 250–280 rpm. The spinner bottles resided in a water bath to maintain the culture temperature, and a five-position magnetic stir plate was located beneath the water bath for agitation. The seed culture was agitated for 24–36 h until the optical density (OD_600_) reached 8 ± 0.5. Production cultures with 10% inocula were grown with the same defined NMS2 medium as the seed culture except using 4× KNO_3_, 2× mL phosphate solution and 4× of trace solution.

A continuous gas delivery and monitoring system was utilized to supply the gas mixture for all batch cultures as described previously [22]. Production cultures were carried out using a Biostat Q-Plus fermentation system (Sartorius, Goettingen, Germany), which was set to control pH at 9.0 ± 0.2 with 2 M NaOH. Dissolved O_2_ was monitored with an Ingold polarographic probe and the agitation speed was manually controlled with a maximum rate of 1000 rpm. The flow rate of the gas mixture (CH_4_ and air) was controlled by a digital on-line mass flow controller (Vögtlin Instruments, Aesch, Switzerland) at 300–900 mL/min depending on the experiment to give a CH_4_/O_2_ ratio of 5:4 to meet safety considerations. To prevent excessive foam formation, a 1% sterile antifoam solution (PPG-PEG-PPG, CAS Number 9003-11-6, Sigma-Aldrich. Co, USA) was added manually during production cultures. The CH_4_, O_2_, and CO_2_ in the off-gas stream from bioreactors were monitored and analyzed by gas sensors (BlueSens, Recklinghausen, Germany) in order to calculate gas uptake/evolution rates (mmol/L/h). All presented results in this study were averaged from experiments carried out in duplicate.

### Analyses

Cell growth was estimated by measuring OD at 600 nm [28]. For cell mass analyses, 10 mL of culture broth was transferred to a pre-weighed centrifuge tube and centrifuged at 5000 rpm for 30 min. After rinsing once with deionized water, the pellet was dried for 24–48 h in a lyophilizer for DCW measurement and lipid analysis [29]. A modified Megazyme procedure (AA/AMG 11/01, AOAC Method 996.11, AACC Method 76.13, ICC Standard Method No. 168) was used to determine the glycogen content of lyophilized bacterial biomass. In summary, 10 to 25 mg of lyophilized cell mass was weighed out into 15-mL plastic centrifuge tubes. To each sample was added 50 μL 190-proof ethanol and 500 μL DMSO, and subsequently the samples were placed in a boiling water bath for 5 min. After boiling, 725 μL of MOPS buffer and 25 μL of thermostable α-amylase were added to each sample and vortexed. Samples were then incubated in a boiling water bath for 12 min, vortexing every 2 min. After 12 min, samples were removed from the water bath and 1 mL of sodium acetate buffer and 25 μL of amyloglucosidase were added and samples were vortexed. Samples were then placed into a 50 °C water bath. After 30 min, samples were removed from the water bath and centrifuged at 3000 rpm for 5 min. An aliquot of each sample was filtered through a 0.2-µm nylon filter and run on an HPLC equipped with a Biorad Aminex HPX-87H column (55–65 °C) and an RID detector. A 0.01 N sulfuric acid mobile phase at 0.6 mL/min was used.

## Results and discussion

### Effects of agitation rates on lipid production by *M. buryatense* 5GB1

Due to the extremely low solubility of CH_4_ and O_2_, which are both required for cell growth and lipid production, the cultivation of methanotrophs can be severely limited by the gas transfer efficiency [30, 31]. Increasing the agitation rate is the simplest way to improve gas transfer efficiency in a continuous stirred tank bioreactor (CSTR), in which a sparger at the bottom of the CSTR delivers gases into the liquid phase [32]. The agitation rate of the stirring blades inside the CSTR is controlled to break down bubbles into smaller sizes in order to increase the gas–liquid interfacial area and to keep them from offgassing quickly, resulting in a better gas transfer efficiency. Two different agitation rates (500 and 1000 rpm) with a gas flow rate of 300 ccm were first tested to gain a basic understanding of the effect of agitation rates on cell growth and lipid production in batch cultures of 5GB1 with continuous feeding of CH_4_ and O_2_. As shown in Table 3, improved cell density (DCW) was obtained under higher agitation rate, which corresponds to a 30% enhancement in cell density compared with previous highest reported data (10.2 g/L) from cultures of 5GB1 [21]. Although a higher lipid content of 9.6% was observed with lower agitation rate, a 30–60% increase in lipid productivity was achieved in cultures with 1000 rpm, which corresponds to 32.3 and 17.9 mg/L/h at 24 and 48 h, respectively. Interestingly, we also found that *M. buryatense* 5GB1 accumulated glycogen under both conditions, but the 1000 rpm agitation rate resulted in a higher glycogen content of 18.7% (Table 3), which is much higher than previously reported in low cell density batch cultures of *M. buryatense* (2.7%) [22]. The high glycogen content obtained from cultures with higher agitation rate indicates a change in the metabolic balance of the cell, such as increased activity of glycogen synthase under this growth condition. This may explain the drop in lipid content from 7.9 to 6.1% as more carbon is devoted to glycogen production.

**Table 3:** Lipid and glycogen (Gly) production by *M. buryatense* 5GB1 with the gas flow rate of 300 ccm under two different agitation rates

Time	500 rpm				1000 rpm			
	DCW, g/L	Lipid, %	Pr_L_, mg/L/h	Gly, %	DCW, g/L	Lipid, %	Pr_L_, mg/L/h	Gly, %
24	5.0 ± 0.3	9.8 ± 0.4	20.4 ± 1.2	2.2 ± 0.5	9.8 ± 1.2	7.9 ± 0.5	32.3 ± 1.9	12.3 ± 1.5
48	6.8 ± 0.5	9.6 ± 0.2	13.6 ± 0.4	1.8 ± 0.1	14.1 ± 0.9	6.1 ± 0.3	17.9 ± 0.8	18.7 ± 1.4

*Pr*_*L*_ lipid productivity

### Enhanced lipid production by *M. buryatense* 5GB1 in high cell density cultivation

**Fig. 3:**
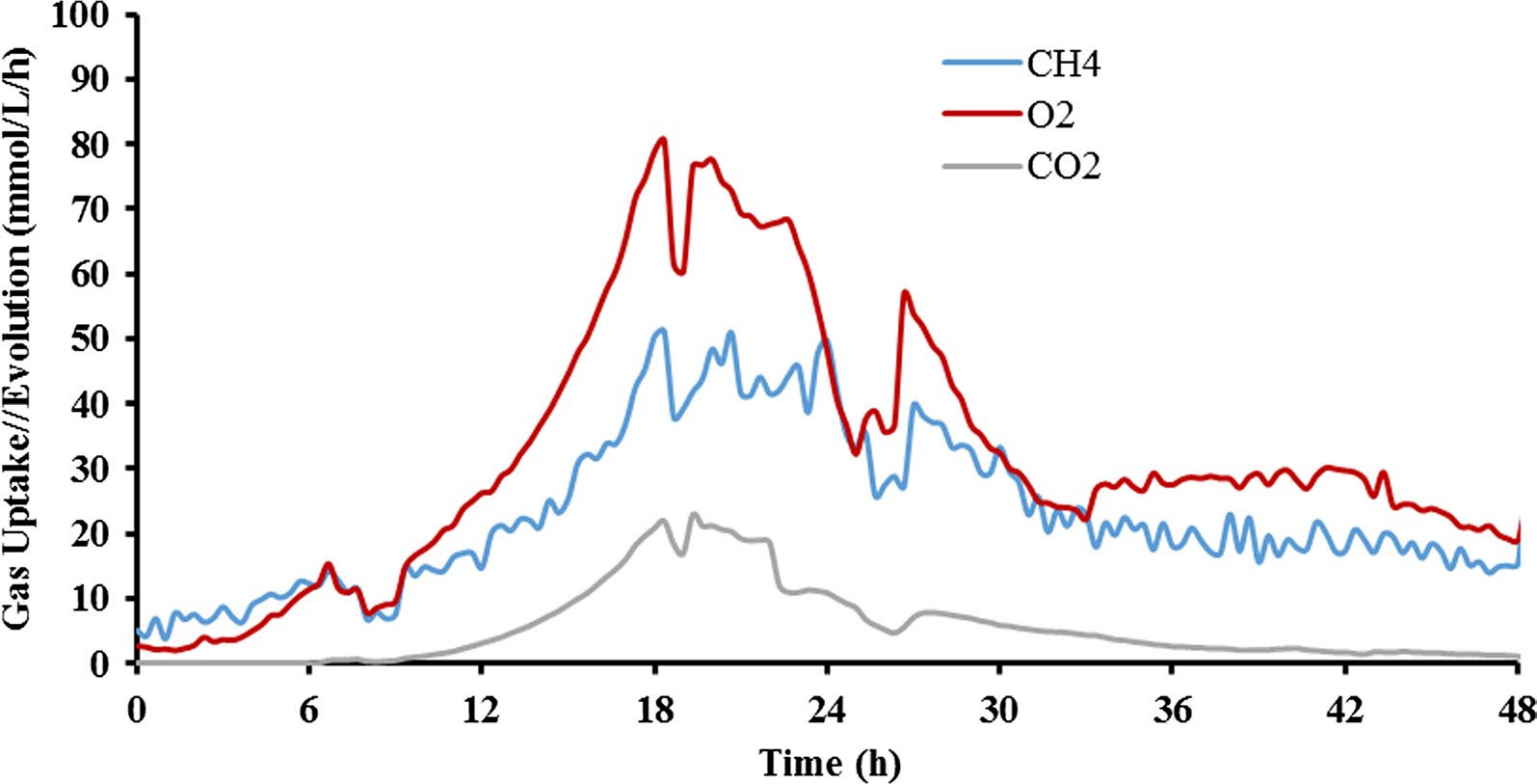
Gas uptake (CH_4_ and O_2_) rate and CO_2_ evolution rate in the cultivation of *M. buryatense* 5GB1 for lipid production

**Fig. 4:**
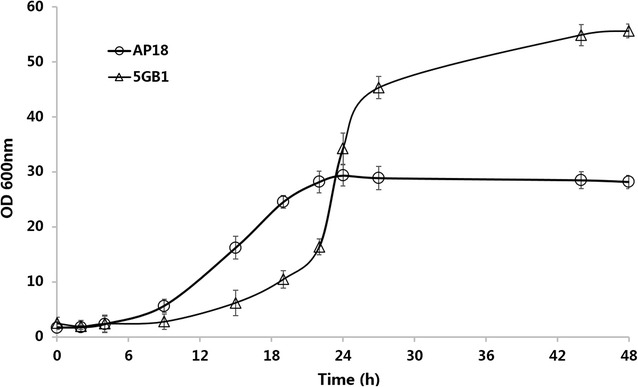
Comparison of growth profile of *M. buryatense* 5GB1 and AP18 in batch cultures

Because lipids produced by *M. buryatense* are in the form of intracellular membranes, the productivity of lipids is a function of both the cellular lipid titer and the cell density in culture, which is in agreement with the aforementioned findings (Table 3). Therefore, high cell density cultivation could be an important strategy to improve methanotrophic cultivation for the liquid fuel production. Both high gas flow rate (900 ccm) and high agitation rate (1000 rpm) were employed to achieve high cell density cultures for the lipid production. As shown in Figs. 3 and 4, an OD of 55 with a maximum specific growth rate of 0.22 h^−1^ was observed during the batch cultures, in which the maximum O_2_ and CH_4_ utilization rates measured were 80.6 and 51.1 mmol/L/h, respectively. After 24 h, the growth rate began to slow down, presumably due to the exhaustion of nutrients in the medium, and the cultures exhibited lower gas utilization rates along with increasing dissolved O_2_ indicating the end of the cultivation.

The highest DCW of 21.4 g/L along with a maximum lipid productivity of 36.1 mg/L/h was accomplished in high cell density cultures of *M. buryatense* 5GB1 (Table 4), which presents a 4-fold cell density improvement and 60% increase in lipid productivity, compared with that observed in the culture agitated at 500 rpm. Although enhanced lipid production performance was obtained, lipid content surprisingly decreased from 6.5 to 4.9% during the cultivation, which is substantially lower than values previously recorded from low cell density cultures [22]. Since the goal of this study is to generate high biomass titer with high lipid content to serve as feedstock for extraction and upgrading to diesel fuel, this approach for high cell density cultures was a limited success. The observed decrease in lipid content may be the result of nutrient depletion in the medium, which would result in decreased expression of membrane-bound particulate methane monooxygenase [33], combined with the accumulation of intracellular glycogen also observed in previous experiments.

**Table 4:** Cell growth, lipid production, and glycogen accumulation in the cultivation of *M. buryatense* 5GB1 and AP18 with the flow rate of 900 ccm and agitation of 1000 rpm

Strain	5GB1		AP18	
Culture time, h	24	48	24	48
DCW, g/L	13.3 ± 1.0	21.4 ± 1.6	11.7 ± 0.4	15.3 ± 1.1
Lipid, mg/L	864.3 ± 37.2	1056.2 ± 41.3	1090.2 ± 30.5	1453.5 ± 54.5
Lipid, %	6.5 ± 0.4	4.9 ± 0.6	9.3 ± 0.3	9.5 ± 0.4
PrL, mg/L/h	36.1 ± 2.2	22.0 ± 1.3	45.4 ± 1.7	30.3 ± 0.9
Gly, %	27.6 ± 3.2	35.9 ± 2.6	0.0	0.0

As shown in Table 4, the glycogen content in *M. buryatense* 5GB1 significantly increased to 35.9% when grown under high cell density culture conditions. These growth conditions cause *M. buryatense* 5GB1 to shift carbon flux to glycogen accumulation at the expense of cell division and membrane synthesis. It has been reported that up to 35% glycogen content can be obtained in cultures of *Methylomicrobium* species using methanol [22, 34]. However, this is the first time that such high glycogen content has been reported for cultures of *M. buryatense* grown on CH_4_ as the sole carbon source. Therefore, although lipid production was improved in high cell density cultures, the great amount of glycogen produced is a significant competing carbon sink that limits lipid biosynthesis [22].

It has been reported that the carbon flux can be significantly enhanced in various microorganisms by limiting synthesis pathways of byproducts [35–37]. The enhancement of lipid accumulation has been demonstrated by blocking the starch synthesis in *Chlamydomonas reinhardtii* under nitrogen limitation condition [38]. Han et al. have recently increased the lipid productivities of *Tetraselmis* sp. up to 30% via regulating the carbon flux from starch synthesis to lipid production [39]. In an attempt to limit production of glycogen, a 5GB1 ∆*glgA1* ∆*glgA2* mutant, which did not produce any glycogen under previously tested low cell density batch conditions [17] was tested and grown under the high cell density culture conditions used in the initial experiments of this study (agitation of 1000 rpm and a flow rate of 300 ccm). However, under this setup, the double knockout mutant is still able to accumulate 6.4% glycogen in a 48-h batch cultivation (data not shown). To address this finding, a new strain was constructed by knocking out the gene encoding for sucrose-6-phosphate synthase (*sps*) in addition to the two glycogen synthase genes, *glgA1* and *glgA2*. This latter gene was targeted because members of this genus of methanotrophs can also synthesize glycogen via sucrose degradation as shown in Fig. 2 [40, 41]. This triple knockout mutant strain was also cured of 5GB1’s native plasmid to enable plasmid-based heterologous expression [17] in other studies [20], resulting in the strain AP18 (5GB1C Δ*glgA1* ∆*glgA2* Δ*sps*).

### Effects of the limitation of glycogen biosynthesis on cell growth and lipid production

For the first time, the effect of limiting glycogen biosynthesis on cell growth and lipid production in high cell density cultures was investigated. The performance of 5GB1 and AP18 was compared in terms of cell density, lipid production, and glycogen accumulation under high cell density culture conditions. As shown in Fig. 4, AP18 reached stationary phase at a lower optical density and had a lower maximum growth rate compared with 5GB1 (the decrease in OD of AP18 was observed after 48 h, data not shown in Fig. 4). However, the glycogen content in AP18 biomass was below detection limits and a consistently high lipid content above 9% was observed throughout cultivation (Table 4), resulting in a 90% increase relative to 5GB1. Finally, a maximum lipid productivity of 45.4 mg/L/h was also achieved by AP18, which presents a 20% improvement over 5GB1 and a 3-fold improvement in lipid production, compared with previous reported data (13.3 mg/L/h) from cultures of 5GB1 [21].

**Fig. 5:**
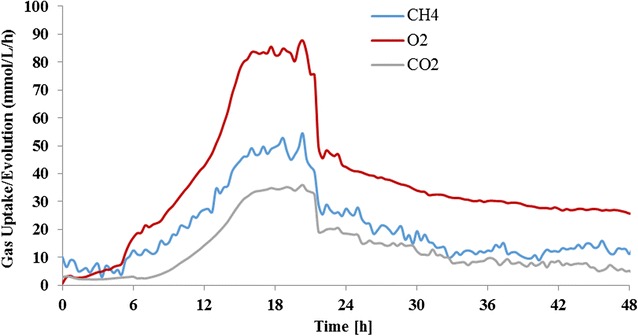
Gas uptake (CH_4_ and O_2_) rate and CO_2_ evolution rate in the cultivation of *M. buryatense* AP18 for lipid production

Although no glycogen was accumulated in AP18, the final DCW of 15.3 g/L from AP18 was surprisingly lower than that seen with 5GB1. After analyzing the gas uptake and evolution in cultures of AP18 (Fig. 5), both specific CH_4_ and O_2_ uptake rates were found to be 45% higher than that in cultures of 5GB1 (Table 5). Interestingly, the specific CO_2_ evolution rate was increased by more than 100% in cultures of AP18 (comparing results in Fig. 5 with those in Fig. 3), which could be due to a need for more reducing power in this mutant. It has been reported that culture conditions can significantly influence the metabolic networks of *Methylomicrobium* species in terms of metabolic networks and NADH production [16, 17], but further investigation will be needed to understand the phenomenon observed in this study. Overall, even though the cell density of AP18 was lower than that of 5GB1 in high cell density culture conditions, the primary metrics for improved biomass production are lipid productivity and content and from this point of view, AP18 clearly exceeded 5GB1 in both of these categories.

**Table 5:** Comparison of cell growth and lipid production by *M. buryatense* under high cell density culture conditions

Strain	5GB1	AP18
DCW, g/L	21.4	15.3
Maximum O_2_ uptake rate, mmol/L/h	80.6	87.8
Maximum CH_4_ uptake rate, mmol/L/h	51.1	54.4
Maximum CO_2_ evolution rate, mmol/L/h	22.9	35.9
Specific O_2_ uptake rate^a^, mmol/gDCW/h	3.8	5.7
Specific CH_4_ uptake rate^a^, mmol/gDCW/h	2.4	3.5
Specific CO_2_ evolution rate^a^, mmol/gDCW/h	1.1	2.3

^a^Calculated by using the maximum gas uptake/evolution rates and the highest DCW from cultures of different strains

### Fatty acid composition of membrane lipids produced by *M. buryatense* using CH_4_ as the sole carbon source

As shown in Table 6, lipids produced by *M. buryatense* 5GB1 in high cell density cultures were composed mainly of myristic acid (C14:0), palmitic acid (C16:0), and palmitoleic acid (C16:1), which is in good agreement with previous reports [21, 23]. The composition of fatty acids showed only minor changes between 5GB1 and AP18. It has been reported that the fatty acid composition can affect the cetane number (CN), which is one of the most significant properties to specify the quality of biofuels for use in a diesel engine [42]. According to the equation developed by Bamgboye et al. [43], CN values of lipids produced by different microbes using various carbon substrates are estimated and listed in Table 6. Compared to other strains, *M. buryatense* showed a relatively high CN value of 75, which is 50% higher than the minimal CN value of biofuel standards set by EU and the US [44]. Our findings suggest that CH_4_-derived lipids from *M. buryatense* can be a very promising alternative source for the liquid fuel production.

**Table 6:** Comparison of fatty acid compositions from different microbes and substrates

Microbe	Substrate	C14:0	C16:0	C16:1	C18:0	C18:1	C18:2	C18:3	CN	References
*M. buryatense 5GB1*	CH_4_	7.1 ± 0.3	16.9 ± 0.5	73.5 ± 0.9	tr	tr	nd	nd	75.1	This work
*M. buryatense* AP18	CH_4_	4.9 ± 0.2	16.8 ± 0.7	75.5 ± 0.6	tr	tr	nd	nd	75.2	This work
*C. vulgaris* (microalga)	CO_2_	nd	24.0	2.1	1.3	24.8	47.8	nd	51.9	[45]
*S. obtusiusculus* (microalga)	CO_2_	nd	30.2	7.3	nd	45.7	nd	12.6	59.5	[46]
*Synechocystis* sp. (cyanobacterium)	CO_2_	nd	52.0	4.0	nd	4.0	14.0	25.0	55.2	[47]
*R. toruloides* (yeast)	Glucose	1.3	25.1	nd	10.4	46.4	10.8	3.4	57.7	[48]

*nd* not detected, *tr* less than 1%, *CN* the cetane number was calculated as described by Bamgboye and Hansen [43]

CN = 61.1 + 0.088X2 + 0.133X3 + 0.152X4 − 0.101X5 − 0.039X6 − 0.243X7 − 0.395X8, where X2 to X8 indicate the weight percentages of methyl esters, as follows: C14:0, C16:0, C16:1, C18:0, C18:1, C18:2, C18:3, respectively

## Conclusions

In this study, high cell density cultures of different *M. buryatense* strains grown with CH_4_ as the sole carbon and energy source were used for the production of the microbial lipid, a potential feedstock for liquid transportation fuels. The highest observed DCW of 21.4 g/L was obtained in batch cultures of 5GB1 with high agitation and flow rate, whereas this high cell density was accompanied by high levels of glycogen and relatively low levels of lipid. One explanation for this could be that at high cell density, 5GB1 metabolism shifts to produce glycogen, and nutrient limitation may also result in down regulation of pMMO synthesis and concomitant reduction in intracellular membrane and membrane lipids [40]. To enhance the carbon flux from CH_4_ to lipids, a mutant (AP18) where glycogen production is abrogated was constructed and used in this research. AP18 provided both high DCW of 15.3 g/L and high lipid productivity of 45.4 mg/L/h. A lipid content of up to 9.3% with undetectable levels of glycogen accumulation was also achieved by AP18, which may therefore serve as a base strain for additional attempts to increase lipid production. Overall, the highest DCW and lipid productivity obtained under high cell density culture conditions present a 2-fold enhancement in cell density and 3-fold improvement in lipid production compared with previous reported data from cultures of 5GB1 [21].

It should be noted that increased CH_4_/O_2_ uptake rate and CO_2_ evaluation rate from AP18 cultures suggest more carbon (CH_4_) and energy are needed to produce lipids by AP18. The resulting low carbon conversion efficiency could be a problem for the economics of the process. As the CSTR approach to cultivation of methanotrophs is highly inefficient and other reactor systems such as bubble columns are better suited for gas-based fermentations [49], it will become important to review the physiological response of 5GB1 and AP18 under high cell density culture conditions to determine if glycogen accumulation will impact lipid production under more commercially relevant conditions. The results of this study expand our understanding on the characteristics of *M. buryatense* under different culture conditions, which provided an insight for the practice of biofixation of CH_4_ for the production of microbial lipid-based bioproducts.

## Abbreviations

CHP: combined heat and power
C14:0: myristate
C16:0: palmitate
C16:1: palmitoleate
C18:0: stearate
C18:1: oleate
C18:2: inoleate
C18:3: linolenate
CN: cetane number
CO_2_: carbon dioxide
DCW: dry cell weight
rpm: rotations per minute
GHG: greenhouse gas
H_2_O: water
HPLC: high-performance liquid chromatography
OD: optical density
CH_4_: methane
O_2_: oxygen
RuMP: ribulose monophosphate
pMMO: particular methane monooxygenase
MDH: methanol dehydrogenase
LDH: lactate dehydrogenase
PDH: pyruvate dehydrogenase
GPD: glycerol-3-phosphate dehydrogenase
GPAT: glycerol-3-phosphate acyltransferase
LPAT: acylglycerol-3-phosphate transferase
PE: phosphatidylethanolamine
PG: phosphatidylglycerol
NADH: nicotinamide adenine dinucleotide (reduced form)
